# Microbiome assembly in thawing permafrost and its feedbacks to climate

**DOI:** 10.1111/gcb.16231

**Published:** 2022-06-20

**Authors:** Jessica G. Ernakovich, Robyn A. Barbato, Virginia I. Rich, Christina Schädel, Rebecca E. Hewitt, Stacey J. Doherty, Emily D. Whalen, Benjamin W. Abbott, Jiri Barta, Christina Biasi, Chris L. Chabot, Jenni Hultman, Christian Knoblauch, Maggie C. Y. Lau Vetter, Mary‐Cathrine Leewis, Susanne Liebner, Rachel Mackelprang, Tullis C. Onstott, Andreas Richter, Ursel M. E. Schütte, Henri M. P. Siljanen, Neslihan Taş, Ina Timling, Tatiana A. Vishnivetskaya, Mark P. Waldrop, Matthias Winkel

**Affiliations:** ^1^ Natural Resources and the Environment University of New Hampshire Durham New Hampshire USA; ^2^ Molecular, Cellular and Biomedical Sciences University of New Hampshire Durham New Hampshire USA; ^3^ EMergent Ecosystem Response to ChanGE (EMERGE) Biology Integration Institute; ^4^ U.S. Army Cold Regions Research and Engineering Laboratory Hanover New Hampshire USA; ^5^ Microbiology Department Ohio State University Columbus Ohio USA; ^6^ Byrd Polar and Climate Research Center Ohio State University Colombus Ohio USA; ^7^ Center of Microbiome Science Ohio State University Colombus Ohio USA; ^8^ Center for Ecosystem Science and Society Northern Arizona University Flagstaff Arizona USA; ^9^ Department of Environmental Studies Amherst College Amherst Massachusetts USA; ^10^ Department of Plant and Wildlife Sciences Brigham Young University Provo Utah USA; ^11^ Centre for Polar Ecology University of South Bohemia Ceske Budejovice Czech Republic; ^12^ Department of Environmental and Biological Sciences University of Eastern Finland Kuopio Finland; ^13^ California State University Northridge Northridge California USA; ^14^ Natural Resources Institute Finland Helsinki Finland; ^15^ Institute of Soil Science Universität Hamburg Hamburg Germany; ^16^ Center for Earth System Research and Sustainability Universität Hamburg Hamburg Germany; ^17^ Department of Geosciences Princeton University Princeton New Jersey USA; ^18^ Laboratory of Extraterrestrial Ocean Systems (LEOS) Institute of Deep‐sea Science and Engineering Chinese Academy of Sciences Sanya China; ^19^ U.S. Geological Survey, Geology Minerals, Energy and Geophysics Science Center Menlo Park California USA; ^20^ Agriculture and Agri‐Food Canada Quebec Research and Development Centre Quebec Quebec Canada; ^21^ GFZ German Research Centre for Geosciences Section Geomicrobiology Potsdam Germany; ^22^ Centre for Microbiology and Environmental Systems Science University of Vienna Vienna Austria; ^23^ Austrian Polar Research Institute Vienna Austria; ^24^ Institute of Arctic Biology University of Alaska Fairbanks Fairbanks Alaska USA; ^25^ Lawrence Berkeley National Laboratory Berkeley California USA; ^26^ University of Alaska Fairbanks Alaska USA; ^27^ University of Tennessee Knoxville Tennessee USA; ^28^ Institute of Physicochemical and Biological Problems of Soil Science Pushchino Russia; ^29^ GFZ German Research Centre for Geosciences Interface Geochemistry Potsdam Germany; ^30^ BfR Federal Institute for Risk Assessment Berlin Germany

## Abstract

The physical and chemical changes that accompany permafrost thaw directly influence the microbial communities that mediate the decomposition of formerly frozen organic matter, leading to uncertainty in permafrost–climate feedbacks. Although changes to microbial metabolism and community structure are documented following thaw, the generality of post‐thaw assembly patterns across permafrost soils of the world remains uncertain, limiting our ability to predict biogeochemistry and microbial community responses to climate change. Based on our review of the Arctic microbiome, permafrost microbiology, and community ecology, we propose that *Assembly Theory* provides a framework to better understand thaw‐mediated microbiome changes and the implications for community function and climate feedbacks. This framework posits that the prevalence of deterministic or stochastic processes indicates whether the community is well‐suited to thrive in changing environmental conditions. We predict that on a short timescale and following high‐disturbance thaw (e.g., thermokarst), stochasticity dominates post‐thaw microbiome assembly, suggesting that functional predictions will be aided by detailed information about the microbiome. At a longer timescale and lower‐intensity disturbance (e.g., active layer deepening), deterministic processes likely dominate, making environmental parameters sufficient for predicting function. We propose that the contribution of stochastic and deterministic processes to post‐thaw microbiome assembly depends on the characteristics of the thaw disturbance, as well as characteristics of the microbial community, such as the ecological and phylogenetic breadth of functional guilds, their functional redundancy, and biotic interactions. These propagate across space and time, potentially providing a means for predicting the microbial forcing of greenhouse gas feedbacks to global climate change.

## Graphical abstract

Microbial communities are active players in climate feedbacks from permafrost thaw. Ecological assembly shapes post‐thaw microbial community composition through deterministic and stochastic processes. In intact permafrost, communities are shaped by selection for permafrost conditions and dispersal limitation. In early thaw, assembly is dominated by dispersal of new members (yellow and orange arrows) and drift (red x’s). Over time, the cumulative impact of selection by abiotic and biotic post‐thaw conditions increases (depicted by filters), and genetic diversification occurs (shown by changes in organism color). Functional guild composition of newly assembled communities impacts post‐thaw ecosystem processes. Artwork by Victor O. Leshyk.

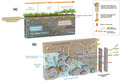

## 1 INTRODUCTION

Microbes are the main players in soil organic matter (SOM) decomposition and greenhouse gas production and consumption (e.g., carbon dioxide [CO_2_], methane [CH_4_], and nitrous oxide [N_2_O]) in thawing permafrost (Box 1). Air temperatures in northern high latitudes are warming faster than the global average (estimates range from 2–4x; Huang et al., 2017; Overland, 2016; Rantanen, 2021), increasing permafrost temperatures at a global scale (Biskaborn et al., 2019). Terrestrial permafrost regions contain an estimated 1400 to 1600 Pg of soil organic carbon (C), twice the amount in the atmosphere (Hugelius et al., 2014; Schuur et al., 2015, 2018). While frozen conditions protect permafrost SOM, warming and thaw make previously frozen SOM available to microbial decomposition, representing one of the largest possible terrestrial feedbacks to anthropogenic climate change (Schuur et al., 2015). The rate and type of greenhouse gas release following permafrost thaw depends on interactions between microbial dynamics, SOM properties, and physicochemical conditions (Graham et al., 2012).

BOX 1 **Permafrost glossary of common terms**

*Permafrost*: Ground that remains at or below 0°C for at least two consecutive years (Van Everdingen, 1998), irrespective of water content.

*Active Layer*: Seasonally thawed soil layer overlying permafrost that provides water and nutrients to vegetation (Van Everdingen, 1998).

*Permafrost‐affected soil*: Soils in subgelic and pergelic climates that include active layer and underlying or nearby permafrost. Gelisols (in the United States Department of Agriculture soil taxonomy or Cryosols in the United Nations Food and Agriculture Organization taxonomy) are defined by having permafrost within 2 m of the soil surface (Keys to Soil Taxonomy | NRCS Soils, 2014). Permafrost‐affection soils are found in a wide variety of ecosystems including forests, tundra, shrublands, grasslands, fens, and bogs, and represent an enormous diversity of soil types, physicochemical properties, and landscape‐level features (Davis, 2001).

*Permafrost zone*: Regions where a portion of the soil column is perennially frozen for more than two consecutive years. Permafrost is distributed continuously toward the High Arctic, and discontinuously at relatively lower latitudes (Brown et al., 2002). It can also be found in Antarctica, high alpine environments, and on the Tibetan Plateau.

*Thaw types*: Permafrost can thaw gradually (i.e., active layer deepening) or abruptly, particularly in ice‐rich areas where melting pockets of ice or ice wedges cause the ground to collapse (e.g., thermokarst, active layer detachments). The formation of thermokarst—a slumping of the land that results from the melting of ground ice—depends on soil environmental conditions, hydrology, soil properties, vegetation, geomorphology, and disturbance (Jorgenson et al., 2013). Active layer detachments are surface landslides where the active layer detaches from the permafrost on hillslopes following warming or heavy precipitation (Lewkowicz, 1990). Depending on the ice content of the permafrost, the aspect of the site and the continued presence or absence of deeper permafrost, thaw can result in saturated or drained conditions on the landscape (Shur & Jorgenson, 2007).

Permafrost is a harsh environment for microorganisms—frozen with limited water availability, highly saline, and with constrained nutrient resources—that changes radically after thaw. Understanding the factors controlling the composition and activity of the microbiome in permafrost following thaw—a disturbance event that has the potential to affect climate on the global scale—is still in its infancy. Molecular analysis has demonstrated that the microbiome of intact permafrost is distinct from the active layer (Mackelprang et al., 2011; Monteux et al., 2018) and that rapid changes to microbiome structure occur following thaw (Barbato et al., 2022; Coolen et al., 2011; Coolen & Orsi, 2015; Mackelprang et al., 2011). Community‐scale ‐omics studies show that microbial communities in the permafrost itself have the potential for highly diverse and complex biogeochemical processes, such as SOM degradation, C fixation, nitrogen (N) metabolism, methanogenesis, and methane oxidation (Hultman et al., 2015; Leewis et al., 2020; Mackelprang et al., 2011, 2017). Although changes to metabolism and community structure have been observed with thaw, it is largely unknown whether post‐thaw assembly patterns are common and predictable among permafrost soils of the world.

Assembly Theory frames the interacting random and nonrandom processes giving rise to community composition (Nemergut et al., 2013; Vellend, 2010). In the Assembly framework, new members are introduced by dispersal and genetic diversification, whereas the abundance of members is determined by selection (i.e., differential survival of lineages due to environmental conditions, including interactions with other organisms), drift (i.e., random variation in the abundance of lineages due to chance), and continued dispersal (Nemergut et al., 2013; Vellend, 2010). These processes are considered stochastic (i.e., random) or deterministic (i.e., nonrandom): drift is understood to be stochastic, selection is viewed as deterministic, and for microorganisms, diversification and dispersal occur along the continuum between stochastic and deterministic depending on the circumstances (Nemergut et al., 2013; Vellend, 2010; Zhou & Ning, 2017) (Box 2). Assembly Theory draws inspiration from population genetics (Vellend, 2010) and is widely and successfully applied to understand how *macro*organismal communities arise after disturbance (e.g., forest gap creation, volcanic eruption, biotic invasion) (Fukami, 2015) and provides an important lens to evaluate microbial responses to disturbance (Dini‐Andreote et al., 2015; Ferrenberg et al., 2013; Nemergut et al., 2013). Soil microbial communities are highly diverse (Thompson et al., 2017), and the degree to which that diversity arises from deterministic factors, such as ecological niche differentiation, or stochastic factors, such as ecological drift, is debated (Knelman & Nemergut, 2014; Nemergut et al., 2013; Tucker et al., 2016). The high spatial heterogeneity in soils creates various niche opportunities for microorganisms over small spatial scales (Trivedi et al., 2017; Wilpiszeski et al., 2019), but also reduces the exchange of genetic information between patches (Cairns et al., 2018; Rillig et al., 2017; Wilpiszeski et al., 2019). The cumulative balance of deterministic and stochastic processes impacts microbiome composition, which in turn affects ecosystem function (Graham & Stegen, 2017; Knelman & Nemergut, 2014). Dominance of deterministic processes results in a community well adapted to environmental conditions; dominance of stochastic processes increases the chance for a high proportion of maladapted taxa, and can decrease some microbiome‐level biogeochemical functions (Graham & Stegen, 2017).

Understanding the assembly processes shaping the post‐thaw microbiome can inform which changes are consequential to community function, and thus to climate feedbacks. If deterministic processes dominate post‐thaw assembly—as is commonly assumed for disturbance in microbial systems (Nemergut et al., 2013)—then classical predictive modeling of carbon and nitrogen cycling may suffice. However, if stochastic processes dominate post‐thaw assembly—as predicted from not only plant communities (Vellend, 2010), but also some microbiome theory and observations (Dini‐Andreote et al., 2015; Ferrenberg et al., 2013; Hughes‐Martiny et al., 2006)—predictive modeling of feedbacks will require explicit representation of stochasticity. Alternatively, some middle ground is possible, whereby the timescale of assembly processes enables roles for both deterministic and stochastic processes. To date, Assembly Theory has been only sparingly applied to permafrost‐associated microbiomes, with a handful of applications to intact permafrost (Bottos et al., 2018; Hu et al., 2015), thawed permafrost (Doherty et al., 2020), the active layer of thawing permafrost landscapes (Feng et al., 2020; Mondav et al., 2017), and the water column of thermokarst thaw ponds (Comte et al., 2016; Moigne et al., 2020). These studies all indicate the importance of both deterministic and stochastic assembly in the microbiomes of permafrost (intact and thawed) and permafrost‐associated soils and waters. In the only study to investigate assembly directly in thawed permafrost, Doherty et al. (2020) found that stochastic assembly is a dominant driver of the composition of the permafrost microbiome in the weeks to months following thaw, but that deterministic assembly plays a larger role in community composition at longer timescales (years to decades). While this only represents one type of permafrost system—a thawing, Little Ice Age era permafrost peatland in subarctic Sweden—these findings suggest that accounting for the contribution of stochastic and deterministic assembly mechanisms is critical to understanding the structure and function of the post‐thaw microbiome. The application of Assembly Theory to studies of the post‐thaw microbiome at a variety of spatial and temporal scales will provide deeper insight into the dominant controls on microbiome composition and function, thus enabling more accurate predictions of permafrost‐climate feedbacks without requiring massive sampling campaigns.

We identify three key controls and the implications of post‐thaw microbiome composition gained from a critical reading of Assembly literature and considering the special case of permafrost thaw. The post‐thaw microbiome composition is influenced by the ecology and physiology of the microorganisms in intact permafrost (Section 2), disturbance intensity (Section 3), and spatiotemporal complexity (Section 4). From this, we predict the cumulative effects of assembly processes influencing the functioning of post‐thaw ecosystems (Section 5).

BOX 2 **A primer on Assembly Theory as it applies to microbial ecology**

While the identities and abundances of resident lineages define a microbiome's composition, community assembly refers to the interacting stochastic and deterministic processes that give rise to the observed composition. The Baas Becking Hypothesis posited that microbial biogeography is explained by selection, such that “Everything is everywhere, but the environment selects” (Baas Becking, 1934). While the preponderance of rare taxa in many microbial communities—diversity's “long tail”—is consistent with this hypothesis, evidence has mounted to support a more nuanced view of how microbiomes arise (Hughes‐Martiny et al., 2006). Nemergut and coauthors (2013) elegantly adapted Vellend's macroorganismal community assembly framework (Vellend, 2010) to microorganisms: new members are introduced by genetic diversification and dispersal, abundances are determined by selection, drift, and continued dispersal (defined and visualized in Box 2 Figure), and these processes exist along a continuum from stochastic to deterministic. While there is a larger pool of potential community members in a landscape, the realized community includes the members that establish after being acted on by these processes, which collectively, results in species turnover (i.e., the loss and gain of lineages in the microbiome composition) over time and/or space. Tools exist to quantify the relative or absolute contributions of stochasticity and determinism (such as variance partitioning, neutral models, null models, and the normalized stochasticity ratio (NST) (Ning et al., 2019; Zhou & Ning, 2017). These tools have different assumptions and focus on different aspects of the microbial community (e.g., phylogenetic or compositional), and have been reviewed recently (Zhou & Ning, 2017).

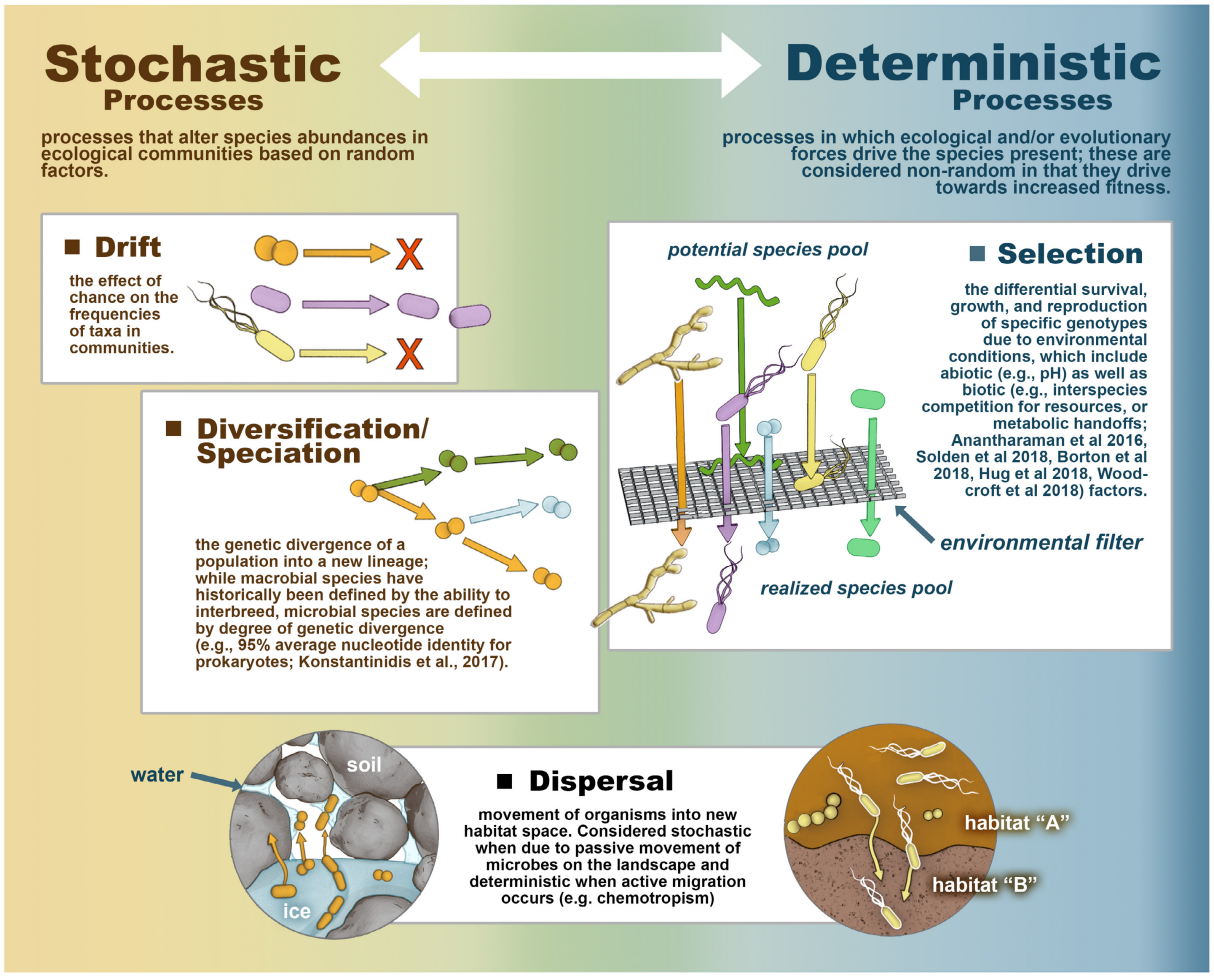

Box 2 Figure. Conceptual diagram of the deterministic and stochastic processes that shape microbiome assembly, on a continuum from purely stochastic (left) to purely deterministic (right) (Anantharaman et al., 2016; Borton et al., 2018; Hanson et al., 2012; Hug & Co, 2018; Konstantinidis et al., 2017; Ruff et al., 2015; Solden et al., 2018; Woodcroft et al., 2018). Diversification in microbes is not purely stochastic since the propensity for it can vary among lineages and conditions (e.g., via receptivity to horizontal transfer, and via the selective relaxation of replication fidelity as in *E. coli*'s mutator strains (Sniegowski et al., 1997)). Artwork by Victor O. Leshyk.

## 2 THE UNIQUE CASE OF PERMAFROST: A COMMUNITY ASSEMBLED IN PERMAFROST SETS THE STAGE FOR HIGH SPECIES TURNOVER AND STOCHASTICITY FOLLOWING THAW

The microbial composition in permafrost is a reflection of multitudinous past events—including the paleoecology during permafrost formation (Holm et al., 2020; Mitzscherling et al., 2019) and the combination of stochastic and deterministic processes that influenced which microbial cells were initially frozen and which survived—and encompasses both active cells thriving in the extreme environment and those in a dormant state. Many permafrost microorganisms show numerous adaptations to sub‐zero, oligotrophic (Mackelprang et al., 2017), high salinity, and cumulative radiation conditions on cellular or subcellular scale including the following: increased membrane fluidity (Ayala‐del‐Rio et al., 2010; Bakermans et al., 2003; Mackelprang et al., 2017); amino acid composition favoring protein flexibility (Raymond‐Bouchard et al., 2018); upregulation of genes, proteins, and transcripts involved in stress response, survival, and maintenance (Coolen & Orsi, 2015; Hultman et al., 2015; Mackelprang et al., 2017); production of cold and osmotic stress proteins (Steven et al., 2006); temperature‐related translation factors (Mykytczuk et al., 2013); increased reliance on horizontal gene transfer, environmental sensing, chemotaxis, and scavenging of detrital biomass (Mackelprang et al., 2017); spore formation and dormancy (Burkert et al., 2019); increased concentrations of intracellular compatible solutes like trehalose, mannitol and sucrose as cryoprotectants (Hoshino et al., 2009; Tibbett et al., 2002); and the capability to seal tissues (e.g., hyphae) broken by freezing (Timling & Taylor, 2012). This greater genetic investment in stress response and maintenance leads to a community with a high proportion of survival‐specialized organisms.

**FIGURE 1:**
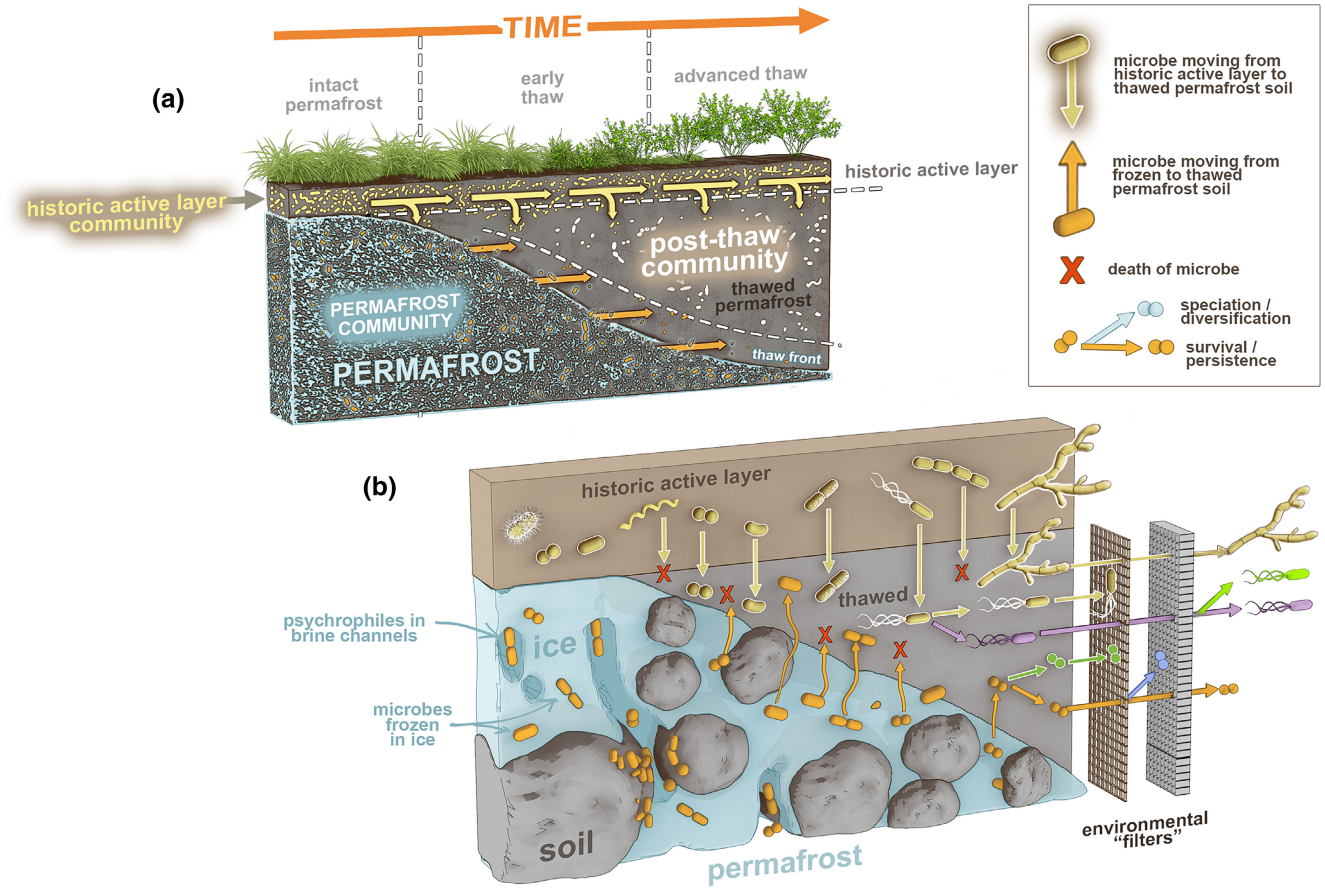
The proposed framework for community assembly processes in a generalized thawing permafrost landscape. In the thawing permafrost landscape through time (a), assembly processes (e.g., drift, diversification, dispersal, selection per Box 2 figure) act on microbial cells from the thawed soil and the overlying active layer, resulting in a post‐thaw community. Within the post‐thaw community (b), acquisition of new members into the species pool is limited by frozen conditions in intact permafrost, where the community is shaped by selection for survival and growth under permafrost conditions. The disturbance of thaw causes immediate disruptions that have a lasting effect on the trajectory of community composition and ecosystem processes. In early thaw stages, the assembly of the post‐thaw microbiome is dominated by dispersal of new members and drift. As time since thaw advances, dispersal and drift continue, while the collective impact of selection by post‐thaw conditions (as depicted by variation in environmental filters over time) builds and genetic diversification occurs (more rapidly, as generation pace increases post‐thaw). Environmental filters may be selective for abiotic factors (like temperature, redox), biotic factors (like interspecies competition for C substrates or predation), or functions. The magnitude of the effect of dispersal, drift, and diversification will depend on time and the intersection of site characteristics and disturbance intensity. The timescales of these processes in natural systems, particularly understudied permafrost systems, is unknown and should be a subject of further research. However, based on studies of transcription (Coolen & Orsi, 2015), community change (e.g., Mackelprang et al., 2011), and assembly (Doherty et al., 2020), it is expected that the immediate effects of the thaw disturbance are realized in minutes to months, and the longer term effects are felt in years to decades. Artwork by Victor O. Leshyk.

Microbial dispersal into permafrost is limited by its frozen state precluding water movement (Bottos et al., 2018; Doherty et al., 2020) (Figure 1), and thus the dominant mechanisms for filling unexploited niches in intact permafrost are diversification via mutation and subsequent environmental filtering (i.e., selection). Furthermore, the initial community structure of the organisms entrained at the time of permafrost formation determines the structure and metabolic potential of the surviving community (Mitzscherling et al., 2019). Drift can be an important contributor to diversity in systems with high selection pressures and low dispersal (Fodelianakis et al., 2021), as confirmed by investigations of assembly in intact permafrost (Bottos et al., 2018; Doherty et al., 2020; Hu et al., 2015). The application of Assembly Theory in *macro*organisms suggests that when organisms remain under the conditions in which the species originally evolved, diversification leads to increased species richness (Vellend, 2010) and a community increasingly adapted to its environment. As such, permafrost microbiomes may exhibit phylogenetic clustering and increasing diversity over centuries and millennia.

Thaw, including permafrost thaw, is one of the most stressful physical transitions known to biological systems, with a phase change from ice to liquid water and changes to the osmotic potential of the soil solution (Gilichinsky et al., 1993). As such, thaw is likely to induce high species turnover. Some permafrost microorganisms become less competitive causing their populations to decrease in abundance or become locally extinct; others may flourish and even break dormancy when conditions, such as decreased salinity, increased temperature, and increased availability of substrates and nutrients, favor their growth. The environmental selection for stress tolerance traits in permafrost microorganisms may put these survival‐specialized permafrost organisms at a disadvantage post‐thaw (Coolen & Orsi, 2015; Hultman et al., 2015; Monteux et al., 2020). When permafrost thaws, new niches open and old niches are altered; thus, the microbiome that diversified over millennia in intact permafrost could be an evolutionary mismatch for post‐thaw conditions, particularly if permafrost‐specialized organisms have a narrow ecological breadth. Ecological breadth refers to how similarly members of a functional guild respond to environmental drivers (i.e., an ecologically narrow guild responds more similarly to a change in abiotic and/or biotic conditions) (Devictor et al., 2010). If a functional guild is ecologically narrow—in other words, has a narrow guild‐level tolerance of some abiotic or biotic conditions, such as temperature, redox, or the presence of particular cross‐feeding partners—then both stochastic and deterministic assembly processes can significantly shape the guild's contribution to the microbiome. Ecologically narrow functional guilds may be more susceptible to stochastic removal or enrichment during post‐thaw assembly, as drift is high and passive dispersal may position cells of that guild in unfriendly habitats (e.g., conditions outside their ecological tolerance). Selection can also have a large guild‐level impact on ecologically narrow guilds, since shifting conditions have a greater potential to environmentally filter the entire guild. In contrast, guilds that are ecologically broad are less susceptible to post‐disturbance assembly impacts on their presence and abundance. An ecologically broad guild is less susceptible to removal or enrichment via environmental filtering at the guild level; however, its individual members are just as susceptible as other lineages, based on their own broad or narrow ecology. Ecologically broad guilds are also less likely to be removed by drift, and if such a guild were missing initially from a community despite its broad ecology, it would be more likely to be imported into a community via dispersal (Graham & Stegen, 2017).

Assembly Theory clarifies that community response to dramatic habitat changes, such as post‐thaw conditions, cannot be considered through the lens of determinism (i.e., selection) alone; stochastic processes also shape outcomes. For example, decreasing salt concentrations during thaw may stress cells, resulting in cell lysis and subsequent declines in microbial biomass (Gilichinsky et al., 1993), but lineage‐specific hardiness—an example of determinism—may be balanced by the random chance of an individual cell's proximity to a mineral surface or local brine concentration—an example of stochastic control on community composition. If a significant proportion of a population survives the initial stress of thaw or if dispersal rates are sufficiently high, there may be a bloom of microbial growth of some founder populations whose establishment may have been rooted in stochasticity. Furthermore, the many rare taxa in permafrost are especially susceptible to local extinction because of their small population sizes (Vellend, 2010)—a fundamentally random process that ultimately shapes the potential trajectory of the microbiome as it reassembles. Thus, the post‐thaw microbiome will depend on the balance of both deterministic and stochastic processes, in ways that depend on microsite environment, lineage genetics (including growth rates) and ecological breadth, and the stochasticity associated with dispersal, drift, and diversification. Collectively, these dictate the importance of permafrost‐adapted lineages in the post‐thaw community and the suitability of the post‐thaw microbiome to the new environmental conditions.

## 3 THE INTENSITY OF DISTURBANCE DURING PERMAFROST THAW ALTERS ASSEMBLY TRAJECTORY

The severity of disturbance influences the assembly of organisms. Chronic, low intensity (i.e., ‘press’) disturbance often increases the importance of selection, while acute, high intensity (i.e., ‘pulse’) disturbance increases the importance of stochastic factors as communities reassemble (Dini‐Andreote et al., 2015; Ferrenberg et al., 2013; Turner et al., 1998), particularly where pre‐disturbance communities are poorly adapted to post‐disturbance conditions. However, disturbance can also interact with dispersal with additive or dampening consequences (e.g., post‐fire soil waxiness decreasing vertical water flow, or drought increasing aeolian transport). Higher dispersal may amplify stochasticity (Graham & Stegen, 2017). Permafrost thaw can be induced by a variety of causes with varying disturbance intensity, from rising temperatures, to increased coastal erosion associated with sea ice declines, to infrastructure development, to wildfire (Schuur & Mack, 2018). Thaw is also not a single condition, but occurs in multiple forms across the landscape, including active layer deepening, active layer detachment, and thermokarst (Kokelj & Jorgenson, 2013) (Box 1). This heterogeneity in thaw morphology is multifactorial, encompassing spatial variation in ice content (Jorgenson et al., 2015), thickness of insulating vegetation cover (Fisher et al., 2016), and vulnerability to hydrological flow paths (Vonk et al., 2019), and leads to variation in the intensity of disturbance and associated impact on the post‐thaw microbiome (Liu et al., 2021).

The effects of disturbance intensity on the microbiome can be investigated by using two morphological end‐members of the disturbance continuum: high intensity represented by abrupt thaw—that is, thermokarst, or the slumping of the active layer and thawed permafrost layers—and lower intensity represented by gradual thaw—that is, active layer deepening within an intact soil profile (Box 1). After abrupt thaw, Assembly Theory suggests that stochastic processes should dominate early assembly due to the high level of disturbance, leading to high variability (Deng et al., 2015) and loss of functional stability in the microbiome in thawed layers. In a collapse scar where thermokarst was generated on a short time span, drift could be particularly important (Figure 1). Thermokarst also likely increases dispersal and colonization of active layer microbes into the recently thawed permafrost, via slumping and mixing at small spatial and short temporal scales and via wind‐ and water‐transport at the landscape scale (Knoblauch et al., 2021). Layering deterministic processes onto these stochastic ones, newly introduced active layer microbes might then be at a selective advantage in circumstances where abrupt thaw increases the availability of labile C, selecting for rapidly growing organisms and excluding permafrost organisms (Ernakovich & Wallenstein, 2015). Although there are currently no studies on disturbance severity and microbial assembly in thawing permafrost systems, parallels can be drawn from knowledge about the impact of wildfire on active layer communities. After wildfire—an abrupt disturbance—decomposition can be reduced long‐term due to decreased microbial biomass (Holden et al., 2016). Microbial biomass (including fungi) was reduced in organic active layer soils even 5 years after fire, decreasing phenol oxidase activity and lignin decomposition (Waldrop & Harden, 2008). High intensity disturbances such as high severity fires can increase the proportions of putative fungal pathogens (Hewitt et al., 2016). In instances where active layer soils are combusted in wildfires (Kasischke & Johnstone, 2005), the size and diversity of the active layer species pool available for dispersal is reduced (Ferrenberg et al., 2013; Hewitt et al., 2013, 2016; Taş et al., 2014), altering which microorganisms are available for dispersal to colonize thawed layers. Similar to wildfire, permafrost thaw can be an abrupt disturbance that decreases microbial function (Monteux et al., 2020). Functioning post‐thaw may be affected long term if the recolonization does not happen quickly.

In gradual thaw with minimal physical disruption (e.g., active layer deepening), we expect that deterministic processes (i.e., selection) will play a larger role than in high intensity disturbance scenarios; however, stochastic processes will still affect assembly, particularly at short timescales (Doherty et al., 2020). Laboratory studies show that permafrost communities diverge within days following thaw, no longer resembling either the permafrost or active layer communities (Barbato et al., 2022; Mackelprang et al., 2011; Martinez et al., 2019). This suggests that new selective pressures impose shifts in the microbiome, although some of the observed changes in the DNA profiles could be due to degradation of preserved DNA (Burkert et al., 2019). Because microbial community composition (Tripathi et al., 2018) and functional potential (Frank‐Fahle et al., 2014) vary by depth in the soil profile, gradual thaw will have a distinct effect on each soil layer. For example, genes for methane oxidation and ammonia oxidation decrease with depth (Frank‐Fahle et al., 2014). A gradual thaw scenario is likely to alter the functional stability and rates of functions as selection acts on the community. Monteux et al. (2018) found that bacterial community structure in permafrost changed with thaw resembling the community structure in the active layer—presumably as microbes dispersed from the active layer and colonized the permafrost layers. Permafrost thaw not only results in compositional changes to the thawed layers, but also in the active layer (Deng et al., 2015; Johnston et al., 2019; Woodcroft and Singleton et al., 2018). For example, changes from more mutualistic to more putatively pathogenic fungal and bacterial taxa in the active layer have been associated with thaw and linked with decreases in plant productivity (Schütte et al., 2019; Seitz et al., 2021). This suggests that thaw, even gradual active layer deepening, alters the pool of microbial functions in active layer soils that can potentially disperse into deeper layers with implications for the functional assembly.

## 4 SPACE AND TIME DIRECT DETERMINISTIC VERSUS STOCHASTIC ASSEMBLY

### 4.1 The effect of time

**FIGURE 2:**
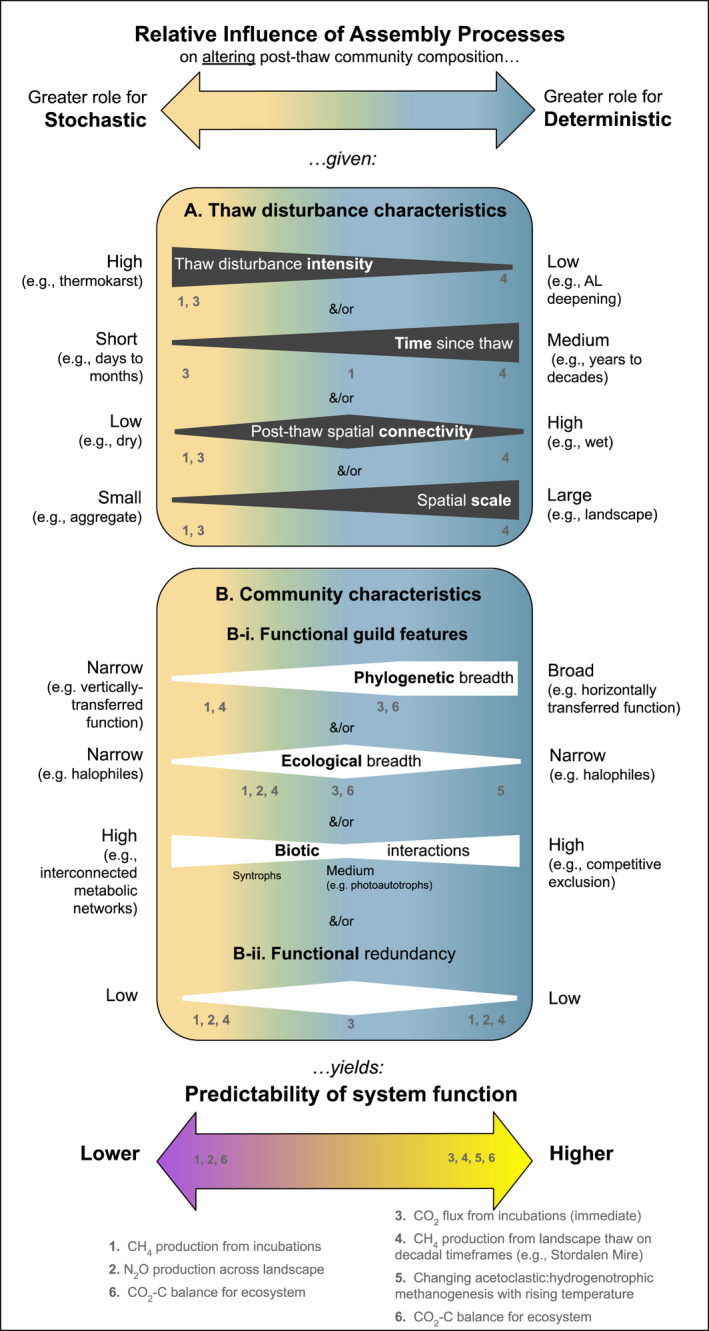
Conceptual framework connecting thaw disturbance and community characteristics to assembly and the predictability of system function. The relative contribution of stochastic and deterministic assembly processes are expected to be impacted by permafrost thaw disturbance characteristics (e.g., time since thaw and thaw disturbance intensity) and functional guild features (phylogenetic and ecological breadth) discussed in Sections 2, 3, 4. Note that all grey and white wedges are independent and are visual representations of the scale of each disturbance or community characteristic (e.g., when disturbance intensity is high the wedge is big, and when it is low the wedge is small). *Phylogenetic breadth* refers to functional guild evolutionary cohesiveness (e.g., narrow for anaerobic methane oxidation, broad for aerobic methane oxidation); phylogenetically narrow, for example, indicates that functional guild members (e.g., those members sharing a trait) are phylogenetically clustered (see Section 4.1). *Ecological breadth* among members of a functional guild refers to how similarly members respond to environmental drivers (i.e., an ecologically narrow guild responds more similarly to a change in abiotic and/or biotic conditions). Notably, these operational concepts depend on the trait and habitat characteristic being considered (e.g., the guild of anaerobic methane oxidizers is phylogenetically narrow and ecologically narrow with respect to oxygen, but not to salinity). See Section 2. The numbers indicate observations in the field or laboratory (references follow), and their placement on the wedges shows the relative contribution of disturbance and functional guild features that affect whether stochasticity or determinism dominate. These characteristics have implications for the predictability of community function (bottom arrow) arising from dominance by stochastic versus deterministic processes, and thus the approaches required to model specific microbiome outputs (such as greenhouse gas emissions). References: 1. McCalley et al., 2014; 2. Drake et al., 2015; Spencer et al., 2015; 3. Allan et al., 2014; 4. See example below; 5. Knoblauch et al., 2018, Ernakovich et al., 2017; 6. Siljanen et al., 2019.

Assembly Theory predicts that stochastic processes should dominate during early assembly following a disturbance that relaxes previous selection (Dini‐Andreote et al., 2015), which is supported by several experimental studies in soils and permafrost (Comte et al., 2016; Mondav et al., 2017). In permafrost, high species turnover is expected immediately following thaw (Figure 1), associated with high rates of drift. Permafrost organisms may be entering the post‐thaw community as permafrost‐adapted organisms or from a state of dormancy; the speed at which they increase metabolic processes following thaw will be a major determinant of early community composition, with important implications for community function. During early succession, the absence of key members can limit processing of organic matter following thaw (Monteux et al., 2020). We expect this to be particularly pronounced for metabolic processes that have narrow phylogenetic breadth—that is, where functional guild members (microorganisms sharing a metabolic trait) are phylogenetically clustered—as there is a greater potential for stochastic processes to remove that entire function. For example, methanogens and nitrifiers are phylogenetically narrow guilds, and even under suitable environmental conditions, the absence of methanogens can limit CH_4_ production (Figure 2, Example 1; Ernakovich et al., 2017; Knoblauch et al., 2018, 2021) and the absence of ammonia oxidizers can limit N_2_O production rates (Figure 2, Example 2; Alves et al., 2019; Siljanen et al., 2019; Monteux et al., 2020; Marushchak et al., 2021). Stochastic factors are particularly impactful for phylogenetically and ecologically constrained guilds (Vellend, 2010). These observations counter the Baas Becking hypothesis that everything is everywhere and demonstrate that stochasticity in microbial membership following thaw can limit ecosystem function.

Whether the immediate and rapid mineralization of permafrost organic carbon to CO_2_ is limited by stochasticity is unresolved (Figure 2, Example 3). No difference in permafrost CO_2_ flux could be attributed to stochastic assembly in one laboratory incubation (Doherty et al., 2020), while in another there were direct links between microbial membership and CO_2_ evolution (Monteux et al., 2020). Inference is confounded by how CO_2_ production is considered as a microbial trait; numerous guilds, with differing phylogenetic and ecological breadth, give rise to CO_2_ production. Substrates and degradation potential are heterogeneously distributed in thawing systems in time and space, with an accumulation of small, easy to decompose molecules under permafrost conditions (Hultman et al., 2015; Leewis et al., 2020; Mackelprang et al., 2011, 2017), non‐uniform distribution of substrate degradation abilities across permafrost layers (Ernakovich & Wallenstein, 2015), and the rapid upregulation of C degradation genes upon thaw (Coolen & Orsi, 2015; Mackelprang et al., 2011).

Functional redundancy has been associated with ecological stability in microbial communities (Biggs et al., 2020) and is likely to play a critical role following permafrost thaw. In plant communities across peatlands, it has been shown that disturbance affects community composition but function stays surprisingly stable, indicating a decoupling of composition and function and suggesting a high degree of functional redundancy (Robroek et al., 2017). Such a pattern appears common in other ecosystems (Burke et al., 2011; Louca & Doebeli, 2016). Indeed, a high degree of functional redundancy could reduce stochasticity in the assembly of CO_2_ degraders. How conserved functions are phylogenetically also depends on whether the function is common, such as respiration which is phylogenetically broad, versus rare, such as methanogenesis or degradation of specific organic compounds which occurs in more closely related taxa (i.e., phylogenetically narrow functional guilds) (Jia & Whalen, 2020; Louca et al., 2018). Aspects such as horizontal gene transfer and loss of function through adaptation further dilute the phylogenetic signal of function (Martiny et al., 2015). Functional redundancy in microbial communities is often inferred based on genomic sequences (e.g., collected from shotgun metagenomics or amplicon‐based analysis of selected functional genes, sensu Louca et al., 2018). While metagenomics is an appropriate tool to investigate microbial community functional potential, it may not be a good measure of functional redundancy because compositional changes after disturbance can alter process rates that are masked by metagenomics (Allison & Martiny, 2008). For these reasons, the relevance of stochasticity to short‐term C cycling after thaw remains an open question and is likely a matter of resolution (of lineages and substrate types) at which the question is examined.

Microbially mediated biogeochemical processes occur at multiple spatial and temporal scales—from cells to soil pores, pedons to plots, and ecosystems to landscapes. The temporal and spatial dynamics of processes such as metabolism, microbial interaction with edaphic properties, and microbiome assembly, means that processes measured at the microbial scale may not necessarily represent emergent biogeochemical processes measured at the ecosystem scale (Hall et al., 2018). For example, at the scale of the cell, transcription and translation are important processes controlling cellular metabolism. These processes are temporally dynamic, changing from second to minute and minute to hour, and demonstrate immediate microbial responses to thaw (Coolen & Orsi, 2015). Studies assessing transcription at only a single point in time may be less informative about the long‐term (e.g., annual or decadal) community turnover and functional response (e.g., diurnal or seasonal biogeochemical processes); however, transcription and translation create proteins which are the engines of organic matter transformation over the longer term and even short‐term insights should not be discounted. Understanding long‐term changes in functional responses can be targeted in long‐term monitoring campaigns or space‐for‐time sampling using methods that have lower temporal resolution (Emerson et al., 2018; Martinez et al., 2019; Woodcroft and Singleton et al., 2018).

### 4.2 The effect of space

Despite the recognition that dispersal is a major determinant of the regional species pool in macroecological communities (Cadotte et al., 2006; Leibold et al., 2004; Vellend, 2010), microbial ecology was long dominated by the notion that microorganisms are not dispersal limited (Baas Becking, 1934). But there is increasing empirical evidence that dispersal plays a role in shaping the composition of microbial communities (Evans et al., 2017). Due to lack of water movement between active layer and intact permafrost, permafrost represents an extreme case of dispersal limitation (Bottos et al., 2018), and as such is a potential model system to test the effects of microbial dispersal limitation on system function. With permafrost thaw, new dispersal pathways are opened between the historic active layer and freshly thawed permafrost.

After permafrost thaws and is incorporated into the active layer, physicochemical conditions become more dynamic as temperature fluctuations cause freeze–thaw cycles, new substrates become available, and changing water content affects oxygen availability. This dynamic state changes the environmental filters that control selection (Box 2 figure). Principally, thaw relaxes the selection related to the frozen environment, but imposes new selective pressures promoting the growth and activity of a new subset of the microbial community. Furthermore, thaw‐induced changes in dispersal and diversification—stochastic processes—bring together new members for biotic interactions. Dispersal may increase post‐thaw microbiome function in the months following thaw (Monteux et al., 2020) because active layer microbial communities are more diverse, grow faster, and degrade more substrates than those in the permafrost (Ernakovich & Wallenstein, 2015).

The dispersal of new community members will play an important role in the assembly of the post‐thaw microbiome, and the rate of dispersal will affect whether the lineages present are a functional match or mismatch for the environment—that is, whether selection can keep pace with dispersal (Graham & Stegen, 2017). If dispersal rates are low relative to selection, selection can act on the new members and create a community well adapted to its environment. However, even in this scenario where selection is more dominant than dispersal, absent lineages may not be ameliorated, leading to decreased rates of key functions. If dispersal rates are high, the community may have high species richness but may not be selected to fill the eco‐evolutionary roles required to maintain maximum ecosystem process rates (Graham & Stegen, 2017). At intermediate rates of dispersal in scenarios with sufficient niche space, selection can act on the new community members, leading to a community with both high diversity and high rates of function (Graham & Stegen, 2017). We expect that these dispersal scenarios, particularly if combined with variable rates of drift across the landscape, can lead to differing founder communities, which will alter the trajectory of the assembly leading to multiple possible outcomes of stable communities (i.e., alternate stable states) in the post‐thaw microbiome (Beisner et al., 2003; Fukami, 2015). This can create a patchwork of microbiomes on the landscape, which will lead to different community outcomes when active layer and permafrost microorganisms interact and assemble the post‐thaw microbiome, particularly given the altered physical and hydrological connectivity that accompanies permafrost thaw. Furthermore, lateral dispersal out of the thawing permafrost and into aquatic systems could alter the composition of the metacommunity, and ecosystem processes, in nearby systems (Heslop et al., 2020).

Biotic interactions—whether inter‐strain, inter‐species, or inter‐kingdom—alter the relative abundance and activity (Aziz et al., 2015; Messan et al., 2020) of microbial community members, attributable directly to selection (e.g., competition for resources, metabolic hand‐offs, and the production of antagonistic compounds) and indirectly to dispersal, drift, and diversification. Focus to date has largely been on bacteria and archaea, but interactions with plants, microeukaryotes, fungi, viruses, and mobile elements (e.g., plasmids, introns, diversity‐generating retro‐elements) will also shape the post‐thaw microbiome and its function. In intact permafrost, antagonistic interactions dominate, whereas mutualistic processes increase in importance following thaw (Messan et al., 2020). Fungi may become a larger part of the post‐thaw microbiome (Coolen et al., 2011), as conditions for saprotrophs specialized for the degradation of complex organic matter (Coolen & Orsi, 2015; Yuan et al., 2018) and mycorrhizal fungi associated with vegetation (Hewitt et al., 2020) become suitable. Because fungi facilitate the decomposition of previously frozen SOM, immobilize and translocate newly released nutrients, and provide a C source that may be important in the priming of ancient permafrost carbon, changes in their relative abundance and activity will alter C cycling at the global scale (Schütte et al., 2019). In addition, viruses infect key C cycling prokaryotes, including methanogens and methanotrophs, making interactions between viruses, archaea, and bacteria an important and under‐investigated consideration when addressing post‐thaw microbiome dynamics (Emerson et al., 2018). Predation and trophic dynamics likely also structure the microbiome following thaw, as has been observed by an increase in heterotrophic flagellates in response to warming in the active layer (Schostag et al., 2019). Microeukaryotes have specific feeding preferences for bacteria, fungi, and viruses (Brown et al., 2020; Geisen et al., 2018, 2021. Thus, interactions between the well‐studied players in the permafrost microbiome (such as bacteria and archaea, and to some extent viruses and fungi) and microeukaryotes, which have been found to be viable in permafrost (Shmakova & Rivkina,^2015^), deserve study becasue they are likely to impact microbiome assembly processes, decomposition dynamics and gas flux post‐thaw.

Plant community succession is a ubiquitous hallmark of permafrost thaw, with drier post‐thaw conditions often leading to increases in woody shrub abundance (i.e., shrubification; Mekonnen et al., 2021) and wetter, inundated scenarios leading to sedges and aquatic vegetation). Plant roots can easily access permafrost soil shortly after thaw (Blume‐Werry et al., 2019), and can provide another means of dispersal for microorganisms. Different plant communities have different rooting patterns and foster distinct microbial communities through symbiotic and antagonistic interactions within the rhizosphere and due to variations in substrate chemistry that microbes use for growth and respiration (de Vries & Wallenstein, 2017; Wallenstein et al., 2007). Plant root exudates can also stimulate microbial metabolism and prime organic matter degradation, a phenomenon observed in laboratory studies of permafrost (Wild et al., 2016). In temperate systems the rhizosphere is a zone where stochastic processes dominate (Beck et al., 2015; Whalen et al., 2021); if applicable to the new rooting zone of plants following permafrost thaw, this will bring another degree of stochasticity into the system. Permafrost thaw releases dissolved N (Finger et al., 2016; Salmon et al., 2018), and the outcome of plant and microbial competition for N is an important determinant of the post‐thaw microbiome (Finger et al., 2016), which can also impact ecosystem‐scale processes such as N_2_O flux (Voigt et al., 2017).

Biotic interactions can have non‐linear responses (e.g., one strain can play the role of repressor or stimulator depending on the ability of the other species present to compete for resources (Aziz et al., 2015)), so the exploration of how biotic interactions alter assembly is important for accurate predictions of microbiome composition. We expect that as time progresses, selection between active layer and permafrost organisms (e.g., by competitive exclusion) likely plays an increasingly larger role in determining microbiome composition, as deterministic processes relating to the physiochemical environment, niche differentiation, stress tolerance, and growth rates act to shape community structure. Permafrost thaw is a unique disturbance to view through an Assembly framework, because the disturbance‐induced increase in stochastic pressures is countered with novel selective pressures which, due to the large biophysical changes that occur with thaw, will not return to the original state. In other words, permafrost thaw is not part of a normal disturbance cycle that the entrained organisms regularly experience. Therefore, observations from temporally and spatially resolved studies are critical to effectively characterize how stochasticity and determinism alter new ecological interactions to influence the successional trajectory of the post‐thaw microbiome.

## 5 THE CUMULATIVE EFFECTS OF ASSEMBLY PROCESSES AFFECT FUNCTIONING OF PERMAFROST ECOSYSTEMS

Assembly processes shape climate feedbacks from thawing permafrost on a global scale by dictating which lineages and functional traits dominate. The predictability of these feedbacks is tied to the relative contribution of deterministic and stochastic processes to the post‐thaw microbiome (Figure 2), where communities dominated by deterministic assembly respond more predictably to changes in the environment and those dominated by stochastic assembly result in less predictability (Knelman & Nemergut, 2014). Stochastically assembled communities are particularly vulnerable to being maladapted to their environment (Graham & Stegen, 2017; Nemergut et al., 2013), which can reduce or eliminate microbially mediated ecosystem processes. Conventional modeling approaches (e.g., frequentist and lacking microbial data) should be sufficient to predict process rates from deterministically assembled communities; in communities where stochastic assembly dominates, modeling approaches might be aided by either explicit representation of the microbiome or by incorporating stochastic assembly mechanisms. We propose that collectively, the level of uncertainty—and thus ease of predictability and modeling—of ecosystem processes arises from the impact of thaw disturbance characteristics (e.g., intensity, time since thaw, spatial connectivity, and scale) and community characteristics (e.g., phylogenetic and ecological breadth, biotic interactions, and functional redundancy) on ecological assembly (Figure 2). We close with examples illustrating links between assembly and function in the context of predictability.

### 5.1 Methane production from permafrost incubations

Methane production can be limited by the low abundance of methanogens (Figure 2, Example 1; Knoblauch et al., 2018; Ernakovich et al., 2017). In a 7‐year laboratory incubation of Pleistocene‐aged permafrost samples, it took years for CH_4_ production to begin, owing to the slow establishment of methanogens in permafrost (Knoblauch et al., 2018); in some instances, methanogens did not establish (and CH_4_ was not produced) until methanogens were seeded from other samples. Interrogating the high variability of methanogen abundance in intact permafrost and the post‐thaw microbiome, we see that there was a high disturbance and spatial isolation layered onto a phylogenetically and ecologically narrow functional guild (Martiny et al., 2015) with low functional redundancy. The post‐thaw methanogen abundance is likely limited by drift and dispersal limitation—stochastic factors that can be particularly impactful for phylogenetically and ecologically constrained guilds (Vellend, 2010)—and environmental filtering—due to the narrow ecological breadth of methanogens and inhospitable conditions. In this instance, predictions of gas flux are aided by information about microbial community structure (particularly the abundance of methanogens), rather than abiotic conditions alone (Ernakovich et al., 2017; McCalley et al., 2014).

### 5.2 Landscape‐level methane flux from older (e.g., decades) thawed permafrost ecosystems

The methane‐cycling functional guilds (i.e., methanogens and methanotrophs) provide a useful case study to consider how changing post‐thaw abiotic conditions (specifically moisture, temperature, and carbon availability) interact with community characteristics to impact community assembly, with global scale implications for the release of CH_4_ to the atmosphere (Figure 2, Example 4). In permafrost landscapes affected by thaw over decadal timescales, the time frame and spatial scale of inference favor the domination of deterministic assembly, as do the community characteristics. The main subguilds of methanogens (e.g., acetoclastic and hydrogenotrophic) differ in their temperature optima (Allan et al., 2014), as well as their reaction rates and substrates: acetoclasts have warmer optima and lower energy yields per CH_4_ molecule produced than their hydrogen‐consuming counterparts (Figure 2, Example 5). Methanotrophs—which oxidize soil methane and thus mediate emissions (Liebner et al., 2009; Winkel et al., 2018)—also show differential responses to increasing temperatures between functional guilds (i.e., type I: those utilizing the ribulose monophosphate [RuMP] vs. type II: serine pathways of biosynthesis) (Knoblauch et al., 2008). Thus, the nature of post‐thaw temperature shifts (with inundated thermokarst producing warmer conditions than gradual deepening in many places) drives environmental filtering of methanogens and methanotrophs, with consequences for system CH_4_ outputs beyond the direct influence of temperature on biochemical reaction rates. Soil redox also dictates the activity and function of methanogens due to the ecological breadth of these functional guilds: fluctuating water availability favors *Methanoflorens* (a hydrogen consumer) (McCalley et al., 2014; Mondav et al., 2014), while static saturated conditions foster *Methanothrix* (an acetate consumer) and *Methanoregula* (a hydrogen consumer) (McCalley et al., 2014; Taş et al., 2018). Lastly, shifts from hydrogenotrophic to acetoclastic methanogens following thaw may in part be driven by changes in substrate availability concurrent with changes in organic matter chemistry (Coolen & Orsi, 2015). This provides further evidence that functional guild features—here the ecologically narrow substrate preferences of methanogens—impact the assembly of the post‐thaw microbiome. In sum, functional guilds that contribute to ecosystem methane flux have varying ecological breadth, and deterministic processes (i.e., environmental filtering by abiotic conditions) will dictate which guilds dominate, and therefore whether the soils are a net sink or source of methane. At decadal or longer timescales, redox state, temperature, and carbon chemistry may be sufficient to model methane flux rather than microbial membership.

### 5.3 Nitrogen cycling

Nitrogen exists in many forms in soil, from organic polymers and monomers to inorganic forms, such as NH_4_^+^, NO_3_^−^, and NO_2_^−^. The conversion of NH_4_^+^ to NO_3_^−^ (i.e., nitrification) is an important control on denitrification rates and plant available N (Siljanen et al., 2019). In thawed permafrost soils, nitrification rates can be depressed due to missing functional groups, only to be alleviated by microbial colonization (Monteux et al., 2020). Nitrifying microbes are phylogenetically broad (Alves et al., 2018), however, in permafrost soils there is evidence that the nitrification pathways are carried out by only a few ammonia oxidizing archaea (Siljanen et al., 2019). The low diversity of ammonia oxidizing archaea in cold ecosystems may function as phylogenetically narrow, even though they are globally broad. Furthermore, environmental filtering in permafrost has resulted in an ecologically narrow functional guild susceptible to stochastic pressures (e.g., local extinction, drift) after thaw. Despite this, there is evidence, although limited, that nitrifier optimal growth rate was well above the average in situ temperature (Alves et al., 2019). Warming has been shown to alleviate limitations on N_2_O production (Salazar et al., 2020), indicating that deterministic processes (i.e., environmental filtering) could become increasingly important following thaw. Indeed, Marushchak et al. (2021) found that shortly after thaw, N_2_O flux was limited, but that within a few years N_2_O rates can increase rapidly concomitant with increasing nitrifier abundance. Functional guilds contributing to N_2_O flux are ecologically, and possibly even phylogenetically, narrow in cold regions. Thus, N_2_O flux may be limited shortly after thaw when stochastic processes tend to dominate (Doherty et al., 2020). Due to the dominance of stochastic processes, predictions of N_2_O flux might be more difficult at early onset of thaw or under high intensity disturbances; as time progresses, determinism increases, making predictions easier.

### 5.4 Ecosystem carbon balance

Net ecosystem C balance is the integrated effect of plant and microbial inputs and outputs (Figure 2, Example 6), with the inputs primarily being plant photosynthesis and carbon storage in biomass and soils and the outputs being plant respiration and microbial CO_2_ production via aerobic respiration and multiple anaerobic respiration pathways (e.g., denitrification, iron reduction). While methanogens also convert CO_2_ to CH_4_ (thus, altering the form of carbon in the balance), we will focus on conversions between organic carbon and CO_2_ (and the inverse) in this example. The characteristics of microbial CO_2_ producers—their broad ecology and phylogeny and high functional redundancy—would suggest that microbial CO_2_ flux is predictable (Figure 2, Example 3; Schimel & Schaeffer, 2012). Ecologically and phylogenetically broad guilds are likely to be present in a microbiome if environmental conditions permit because they share similar interactions with stochastic processes, in that they have low susceptibility to removal by drift and more likelihood of introduction by dispersal. Phylogenetic and ecological breadth do not always co‐vary (as the guild‐defining trait may be horizontally transferred but ecologically constrained or solely vertically transferred and thus limited to a single clade whose members exhibit individually or collectively broad ecological tolerances), but in this case they do (Martiny et al., 2015). The extent to which a function's phylogenetic and ecological breadth are consequential to ecosystem function is dependent on the redundancy of the function within the post‐thaw microbiome, which is high in the case of microbial CO_2_. But the interaction between disturbance characteristics and characteristics of other functional guilds in the microbial community adds uncertainty to linked processes at the ecosystem scale. For example, while plant carbon fixation (i.e., photosynthesis) is well‐represented in models, the plant available N is limited by microbial processes (see Figure 2, Example 2). Because stochasticity is inherent in the microbial N cycle in thawed permafrost, even ecosystem C balance can be hard to predict. Applying Assembly Theory to better understand microbial functional guilds responsible for coupled biogeochemical processes in permafrost‐affected soils would inform how microbiomes assemble and constrain the uncertainty in the ecosystem carbon balance following thaw.

## 6 CONCLUSIONS

Permafrost is thawing at an alarming rate, creating a globally replicated experiment in microbiome assembly dynamics following ost‐disturbance. The post‐thaw microbiome represents a critical unknown in predictions of permafrost C decomposition, greenhouse gas release, and terrestrial feedbacks to the climate. While a handful of studies have observed changes in microbiome structure, activity, and function after thaw, a unified theoretical framework should be employed to understand—and more importantly predict—how microbiomes assemble and function following thaw. Assembly Theory is a robust framework that can be employed to *understand* the factors that dictate which microorganisms are present and active upon thaw and *predict* how permafrost‐affected systems respond to warming, and thus impact the global climate system. Using the lens of Assembly Theory, we highlight the importance of stochastic mechanisms—particularly drift and dispersal—in shaping the initial microbiome in thawing permafrost, and an increasing contribution of deterministic processes—particularly selection via filtering for new environmental conditions and biotic interactions—over time.

Future studies of the post‐thaw microbiome should not only address questions of community composition, but also should leverage the power of this Assembly framework and build off earlier successes applying Assembly Theory to intact (Bottos et al., 2018; Hu et al., 2015) and thawed permafrost landscapes (Comte et al., 2016; Doherty et al., 2020; Feng et al., 2020; Moigne et al., 2020; Mondav et al., 2017). The ability to predict post‐thaw microbiomes and their impact on ecosystem function can be advanced by interrogating how microbiome assembly emerges from the impact of different thaw scenarios on microbial community characteristics (e.g., phylogenetic and ecological breath, biotic interactions, and functional redundancy) in light of novel post‐thaw hydrological regimes and plant–microbiome interactions, as well as human interactions with Arctic landscapes. Efforts using Assembly Frameworks to understand the factors underpinning microbiome composition and its consequences for function will be advanced by the large‐scale, high‐resolution molecular and biogeochemical datasets now becoming available for permafrost‐affected microbiomes (Burkert et al., 2019; Mondav et al., 2014; Woodcroft et al., 2018).

Furthermore, future efforts should make use of phylogenetic, metabolic, and trait‐based tools (Dini‐Andreote et al., 2015; Horner‐Devine & Bohannan, 2006; Shaffer et al., 2020; Stegen et al., 2012; Tucker et al., 2004) to interrogate assembly processes, probe the environmental and biological filters that contribute to deterministic assembly, and put bounds on the effect of time and space. Large unknowns in how deterministic processes structure the community, such as niche differentiation and competition, may be better understood from experimental incubations, but are seldom translated to the field (Louca & Doebeli, 2016). Stochasticity in assembly needs to be recognized by microbial and ecosystem ecologists, as well as Earth System modelers, by not only representing dispersion in microbial and process rate data, but also by understanding whether—and which—random processes underlie the variation in responses. The framework provided by Assembly Theory—and outlined here by an international group of permafrost microbial ecologists—should ignite the design of a global observation and modeling effort to observe the changing permafrost microbiome, as its findings could have far‐reaching implications for understanding, and designing global policy around, permafrost–climate feedbacks in a warming world.

## AUTHOR CONTRIBUTIONS

JGE conceived of the manuscript and all authors contributed equally to original outline and literature review. Concepts were refined by JGE, RAB, VIR, CS, REB, SJD, and EDW with co‐author input and substantial edits.

## CONFLICT OF INTEREST

The authors declare no conflicts of interest.

## Data Availability

Data sharing not applicable ‐ no new data generated, or the article describes entirely theoretical research.
